# Transcriptome analysis of tongue cancer based on high-throughput sequencing

**DOI:** 10.3892/or.2020.7560

**Published:** 2020-03-23

**Authors:** Mingming Tang, Wencheng Dai, Hao Wu, Xinjiang Xu, Bin Jiang, Yingze Wei, Hongyan Qian, Liang Han

**Affiliations:** 1 Department of Head and Neck Surgery, Nantong Tumor Hospital, Nantong, Jiangsu 226361, P.R. China; 2 Department of Otorhinolaryngology Head and Neck Surgery, Affiliated Hospital of Nantong University, Nantong, Jiangsu 226001, P.R. China; 3 Department of Clinical Pathology, Nantong Tumor Hospital, Nantong, Jiangsu 226361, P.R. China; 4 Key Laboratory of Cancer Research Center Nantong, Nantong Tumor Hospital, Nantong, Jiangsu 226361, P.R. China

**Keywords:** tongue cancer, RNA sequencing, Gene Ontology analysis, Kyoto Encyclopedia of Genes and Genomes analysis, phosphoinositide 3-kinase-Akt

## Abstract

Tongue cancer is one of the most common types of cancer, but its molecular etiology and pathogenesis remain unclear. The aim of the present study was to elucidate the pathogenesis of tongue cancer and investigate novel potential diagnostic and therapeutic targets. Four matched pairs of tongue cancer and paracancerous tissues were collected for RNA sequencing (RNA-Seq), and the differentially expressed genes were analyzed. The RNA-Seq data of tongue cancer tissues were further analyzed using bioinformatics and reverse transcription-quantitative PCR analysis. The sequenced reads were quantified and qualified in accordance with the analysis demands. The transcriptomes of the tongue cancer tissues and paired paracancerous tissues were analyzed, and 1,700 upregulated and 2,249 downregulated genes were identified. Gene Ontology analysis uncovered a significant enrichment in the terms associated with extracellular matrix (ECM) organization, cell adhesion and collagen catabolic processes. Kyoto Encyclopedia of Genes and Genomes analysis demonstrated that these differentially expressed genes were mainly enriched in the focal adhesion pathway, ECM-receptor interaction pathway, phosphoinositide 3-kinase (PI3K)-Akt pathway, and cell adhesion molecules. Comprehensive analyses of the gene tree and pathway network revealed that the majority of cell cycle genes were upregulated, while the majority of the genes associated with intracellular response, cell adhesion and cell differentiation were downregulated. The ECM-receptor interaction, focal adhesion kinase (FAK) and PI3K-Akt pathways were closely associated with one another and held key positions in differential signaling pathways. The ECM-receptor, FAK and PI3K-Akt signaling pathways were found to synergistically promote tongue cancer occurrence and progression, and may serve as potential diagnostic and therapeutic targets for this type of cancer.

## Introduction

Oral cancer is a prevalent malignant tumor. Owing to the frequent mechanical stimulation, the incidence of tongue squamous cell carcinoma ranks first among oral cancer cases, and its incidence continues to increase (1,2). The incidence of tongue cancer is obviously higher among older patients, as the majority of the patients are aged ~60 years (3). Oral cancer is characterized as highly malignant, with high rates of local recurrence and cervical lymph node metastasis (4,5). Currently, surgery combined with postoperative radiotherapy and chemotherapy is the preferred treatment for tongue cancer (6). Due to the short-term recurrence and poor therapeutic efficacy, oral cancer has a dismal prognosis and severely affects the life quality of the affected patients (7). The 5 year survival rate of oral cancer is reported to be ~50% (8). The symptoms of early-stage oral cancer are atypical and they are often mistaken for a bite or a mild stabbing pain. Consequently, several patients with oral cancer already have intermediate- or advanced-stage disease at initial diagnosis and, thus, have missed the optimal window for treatment. Tongue cancer must be predicted and diagnosed as early as possible; thus, it is crucial to elucidate the molecular mechanisms underlying its etiology and pathogenesis.

Bioinformatics analyses of gene expression profiles have been extensively performed in recent years. As an assemblage of RNAs, the transcriptome is mainly transcribed from specific tissues or cells at a certain phase or functional state. Analyzing transcriptome data enables assessing overall gene function and structure, thereby elucidating the potential molecular mechanisms underlying pathological conditions, and has previously been applied extensively in cancer research (9). Non-coding RNAs (ncRNAs) are a type of RNA transcribed from DNA that lacks protein-encoding ability. Critical functions of ncRNAs have been highlighted in almost all aspects of cancer progression (10,11).

Transcriptome analyses have been well documented in cancer research; however, few transcriptome analyses have been reported for tongue cancer, and traditional *in vivo* experiments or single-gene studies are currently preferred. To the best of our knowledge, this is the first study to date to investigate differentially expressed mRNAs and ncRNAs in tongue cancer and paracancerous tissues via transcriptome analysis, in the hope that the findings may help identify potential targets for the diagnosis and clinical treatment of early-stage tongue cancer.

## Materials and methods

### 

#### Subjects

The present study was approved by the Medical Ethics Committee of Nantong Municipal Tumor Hospital (approval no. 2018037). Tongue cancer tissues and matched paracancerous tissues (located ~3 cm from the cancerous tissues) were surgically extracted from 4 patients with tongue cancer (cases 1–4, Table I). A total of 3 men and 1 woman were enrolled, with a mean age of 67 years. Pathologically, 3 of the patients had well-differentiated squamous cell carcinoma (n=2 with T3N2bM0, stage IVb and n=1 with T2N2cM0, stage IVb), and 1 patient had moderately differentiated squamous cell carcinoma (T2N0M0, stage II). Based on the tongue cancer subtypes, 3 patients had ulcerative infiltrating tongue cancer, and 1 had exophytic tongue cancer. Samples used for reverse transcription-quantitative PCR (RT-qPCR) were extracted from 20 patients, whose data are shown in supplementary Table SI. The mean age of these patients was 61.6 years (range, 51–73 years).

#### Hematoxylin and eosin (H&E) staining

Tongue cancer and paracancerous tissues were used for morphological observation with H&E staining as previously described (12). All the following steps are performed at room temperature. In brief, the tissues were fixed in paraformaldehyde for 4 h, dehydrated in graded concentrations of ethanol (70 to 100%, 1 min each), permeabilized in xylene for 5 min, and embedded in paraffin. After embedding, the tissues were cut into 4 µm sections, washed in descending concentrations of ethanol to remove the xylene (100 to 75%, 1 min each, with a final wash in water), stained with hematoxylin for 5 min followed by washing in water, and then stained with eosin for 2 min. The sections were observed and images were captured at a magnification of ×100 using the Olympus IX71 inverted microscope (Olympus Corporation).

#### cDNA library construction and sequencing

Total RNA was extracted from the tongue cancer and paracancerous tissues using TRIzol reagent (Thermo Fisher Scientific, Inc.). The RNA concentration and purity were determined using an ultraviolet spectrophotometer (RAY-757CRT; Raylabel Instrument Co., Ltd.). The cDNA library was constructed and sequenced as previously described (13). In brief, as much rRNA as possible was removed to obtain the purified RNA. Subsequently, mRNA and ncRNA were separated by poly (A) splicing. The RNA was randomly sliced into short fragments that were used as templates. The first-strand cDNA was synthesized alongside 6-bp random primers. The second-strand cDNA was synthesized using a commercial kit (Takara Biotechnology Co., Ltd.) following the manufacturer's instructions. After purification, end-repair, A-tailing and addition of adaptor sequences, the cDNA was fragmentated using uracil glycosylase.

cDNA fragments were subjected to PCR amplification, and the complementary cDNA library was constructed. ncRNAs and mRNAs were sequenced using the high-throughput and high-sensitivity HiSeq 2500 sequencing platform (Illumina, Inc.). Sequenced data were analyzed and processed to dynamically remove the sequence fragments at the 3′-end and low-quality fragments using Trim Galore −0.6.5 software (Babraham Institute). Finally, Fast-QC version 0.11.8 (Babraham Institute) was used to evaluate the quality of the preprocessed data.

#### Comparison with reference sequences

RNA-Seq data were compared with the reference database (GRCh, version 38) using Hisat2 software version 2.1.0 (Johns Hopkins University). Data were mapped with a 56-kb index by genetic fate mapping. Reads were quickly and accurately located on the genome to obtain the genomic structure of the sequencing data.

#### Analysis of differentially expressed genes

Gene expression was quantified via reads per kilobase per million mapped reads (RPKM), which was calculated using Htseq software version 0.9.0 (GitHuB, Fabio Zanini, University of New South Wales, Sydney) as follows: RPKM = total exon reads/[mapped reads × exon length (kb)]. Fold changes (FCs) of the differentially expressed genes in the tongue cancer and paracancerous tissues were calculated. Genes with log_2_ FC >1 or <-1 and a false discovery rate ≤0.05 were selected.

#### Gene Ontology (GO) analysis

GO analysis revealed significant enrichment of the terms associated with biological processes, molecular functions and cellular components. GO terms were assigned based on differentially expressed mRNAs and host genes with significantly different circRNAs. The P-value of each GO term was calculated using Fisher's exact test, and P<0.05 was considered to indicate statistically significant differences.

#### Kyoto Encyclopedia of Genes and Genomes (KEGG) analysis

Pathway annotation of differentially expressed genes was performed using the KEGG database. The P-value of each pathway involved was analyzed using Fisher's exact test based on hypergeometric distribution. P<0.05 was considered to indicate statistically significant differences.

#### RT-qPCR

Tongue cancer and paracancerous tissues from each patient were considered as a group of samples to verify the changes of cancer-related genes by RT-qPCR. The total sample size was 20, and basic patient information is provided in Table SI.

According to the results of gene expression and signaling pathway analysis, 10 genes that were significantly different and were associated with cancer were selected for RT-qPCR. Their primers are listed in Table II (Sangon Biotech, Co., Ltd.). The reverse transcription kit used was PrimeScript™ RT reagent Kit with gDNA Eraser (Takara Biotechnology Co., Ltd.), first at 42°C for 2 min with gDNA eraser and buffer 1, and then at 37°C for 15 min and 85°C for 5 sec with enzyme mix, RT primer and buffer 2. qPCR (Takara Biotechnology Co., Ltd.) was performed at 95°C for 10 min, then at 95°C for 15 sec, 60°C for 30 sec and 72°C for 40 sec for 40 cycles, with a final step at 72°C for 5 min. The reactions were set up in 96-well format Microseal PCR plates (Bio-Rad Laboratories, Inc.) in triplicates.

#### Signaling pathway network

The signaling pathway networks were depicted using Cytoscape 3.4.0 software (Institute for Systems Biology). Each pathway network was depicted based on the pathway terms, and those with P<0.05 were analyzed by KEGG.

#### Statistical analysis

The data are presented as mean ± standard error of the mean. RNA-sequencing data were obtained from 4 independent experiments, while RT-qPCR data were obtained from 20. These data were mainly compared by log_2_FC to explain the upregulation of gene expression in cancer tissues.

## Results

### 

#### Tissue characteristics and morphology

**Figure 1.**
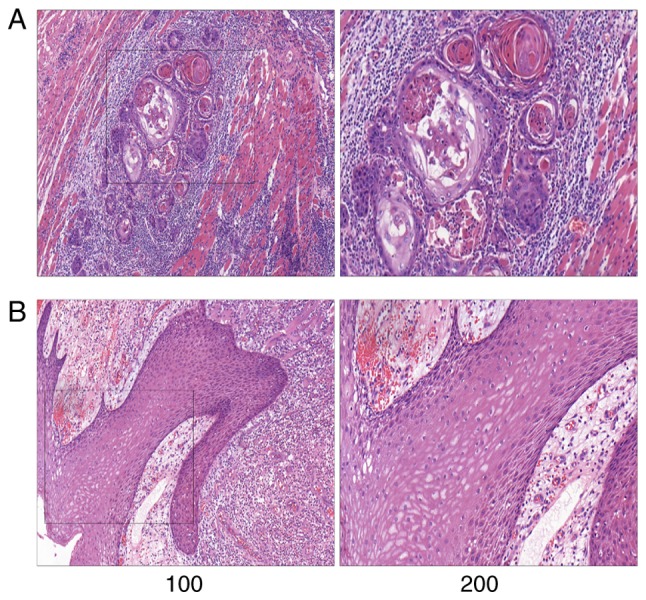
Representative pathological images of tongue cancer cases. (A) Tongue carcinoma tissue; (B) normal tissue. Hematoxylin and eosin staining; magnification in the left and right panels, ×100 and ×200, respectively.

Representative pathological images are shown in Fig. 1. The normal tongue mucosa is composed of stratified squamous epithelium, while the tongue cancer tissues were mostly highly differentiated, accompanied by large amounts of extracellular keratinization and intercellular bridges.

#### Mass analysis of the RNA-Seq data

Transcriptome sequencing and data filtering were performed on 4 matched pairs of tongue cancer and paracancerous tissues. After removing adaptor sequences, the acquired data were compared with the reference genome. Large data sequences and high unique mapping rates of the samples indicated that the quantity and quality of the sequenced data were in accordance with the requirements. The raw data have been uploaded to the NCBI online database, and the accession number is GSE143950.

#### Genome composition of the sequenced samples

**Figure 2.**
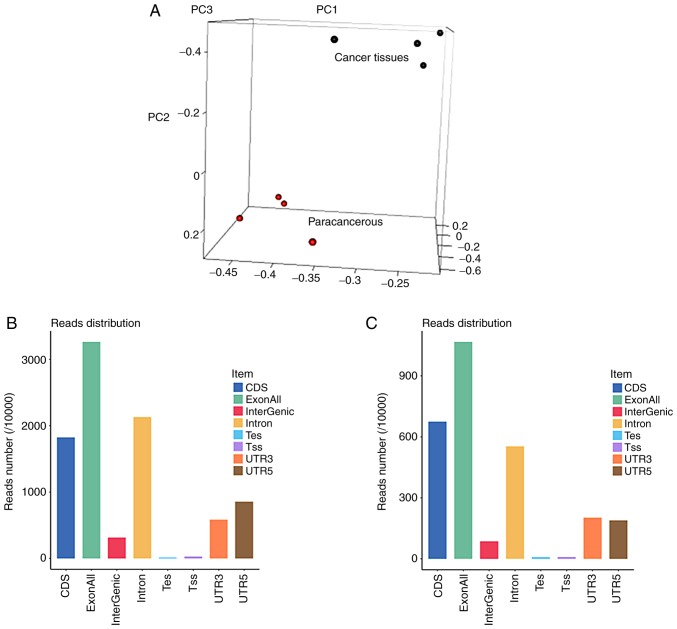

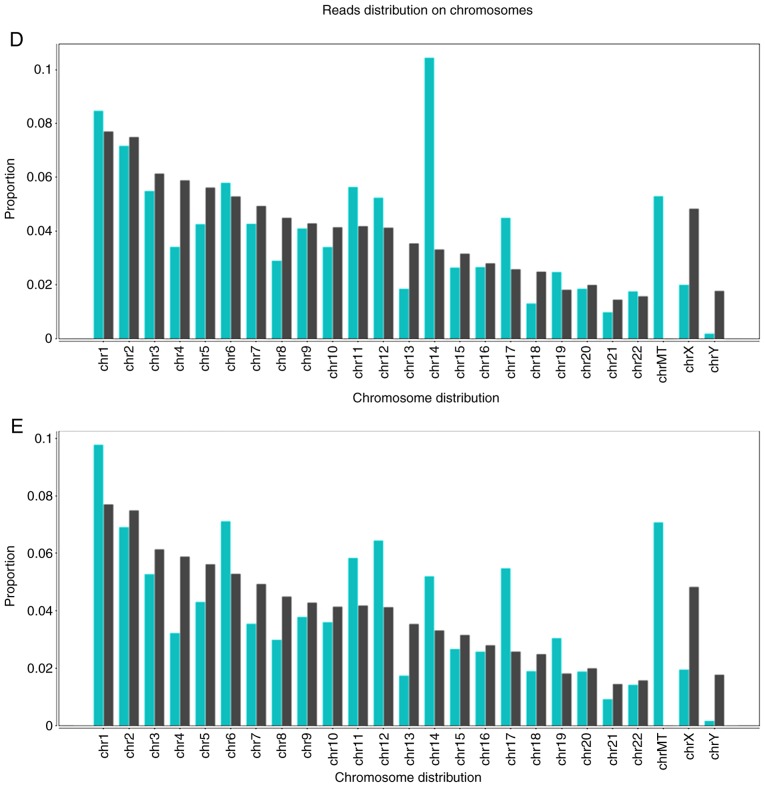

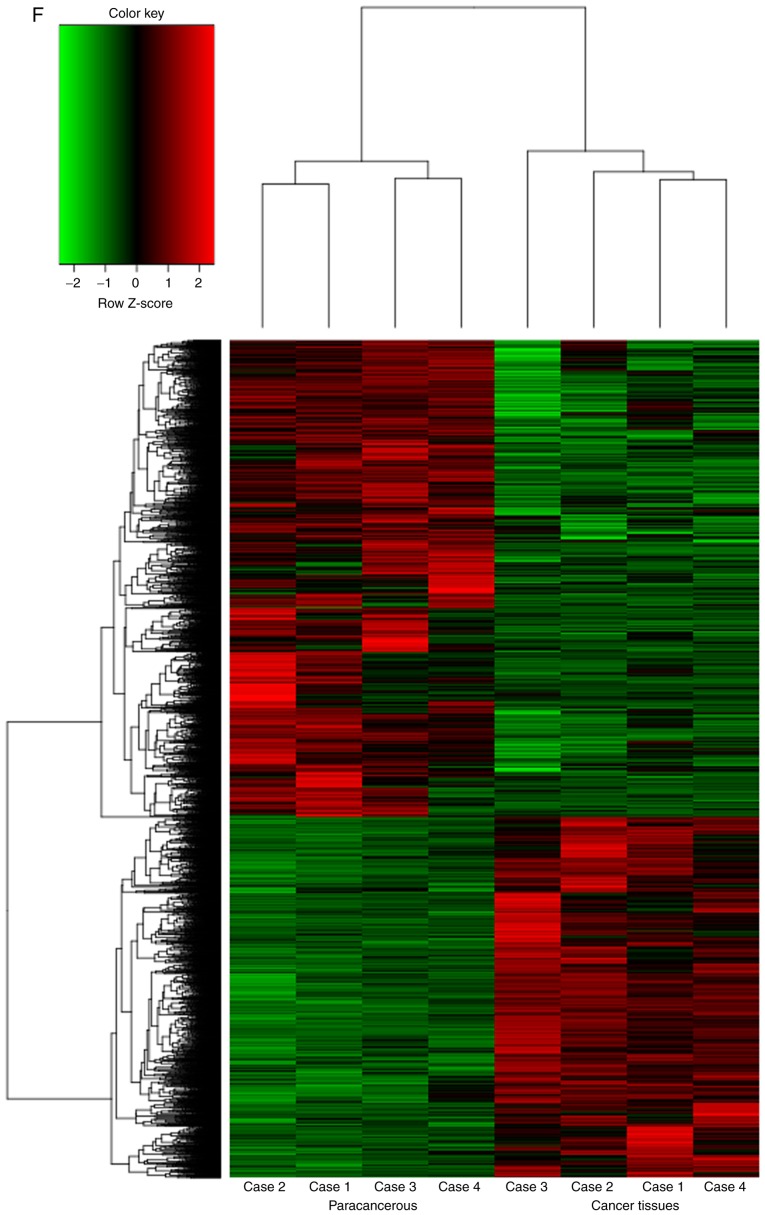
Gene expression. (A) 3D Principal component analysis. (B) Gene reads distribution of tumor tissues. (C) Gene reads distribution of paracancerous tissues. (D) Gene distribution on chromosomes of tumor tissues. (E) Gene distribution on chromosomes of paracancerous tissues. Gene expression. (F) Heatmap of each patient's gene expression.

Principal component analysis (Fig. 2A) revealed a good clustering effect for both the cancerous and adjacent tissues. The genetic composition of the samples in the exons, introns and intergenic regions is shown in Fig. 2B. Abundant RNA transcripts were detected in both exons and introns, suggesting the presence of numerous ncRNAs. The gene composition and distribution on the chromosomes is shown in Fig. 2C. Compared with the paracancerous tissues, Chr.14 was upregulated, while Chr.MT was downregulated in tongue cancer tissues. The gene expression in each patient's cancerous and paracancerous tissues is shown in Fig. 2D.

#### Analyses of differentially expressed genes

**Figure 3.**
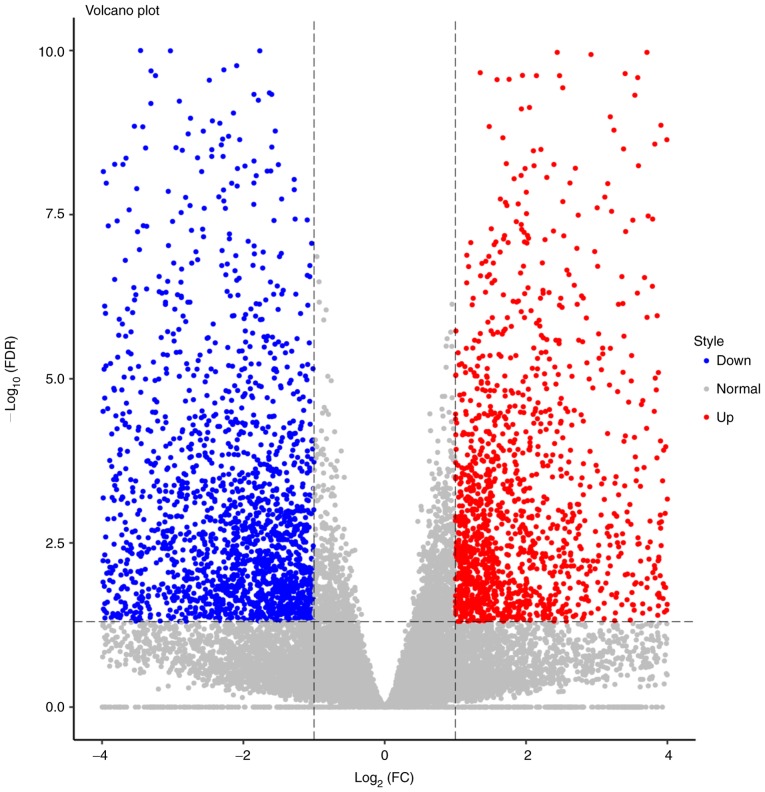
Volcano plot of differential gene expression.

Gene expressions were quantified via RPKM. Expression abundances were calculated and depicted as volcano plots. In total, 24,582 genes were examined, of which 1,700 were upregulated and 2,249 were downregulated (Fig. 3). The top 20 differentially expressed mRNAs and ncRNAs are listed in Table III. The top 4 genes were MMP13 (log_2_FC=11.35), KRTAP13-2 (log_2_FC=−10.52), KRT36 (log_2_FC=−10.07) and S100A7A (log_2_FC=10.00). The heatmap revealed that the trend in gene expression change was consistent in all 4 patients, and the differences between the cancerous and paracancerous tissues were statistically significant.

#### GO analysis

**Figure 4.**
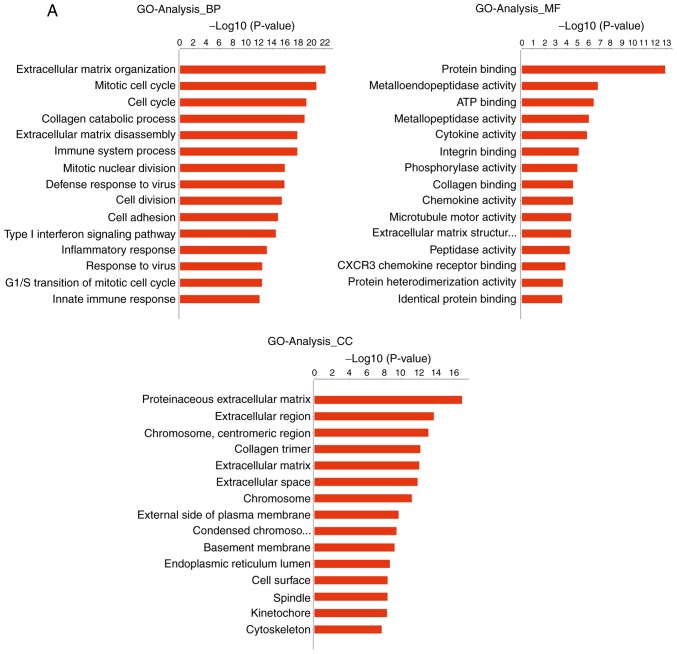

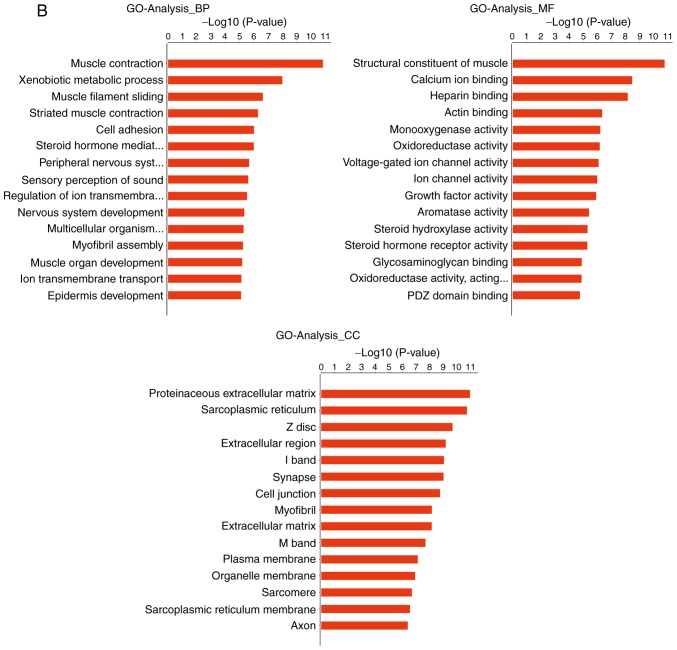
GO terms of differential genes in cancer and normal tissues. (A) Upregulated; (B) downregulated. GO, Gene Ontology; BP, biological process; MF, molecular function; CC, cellular component.

**Figure 5.**
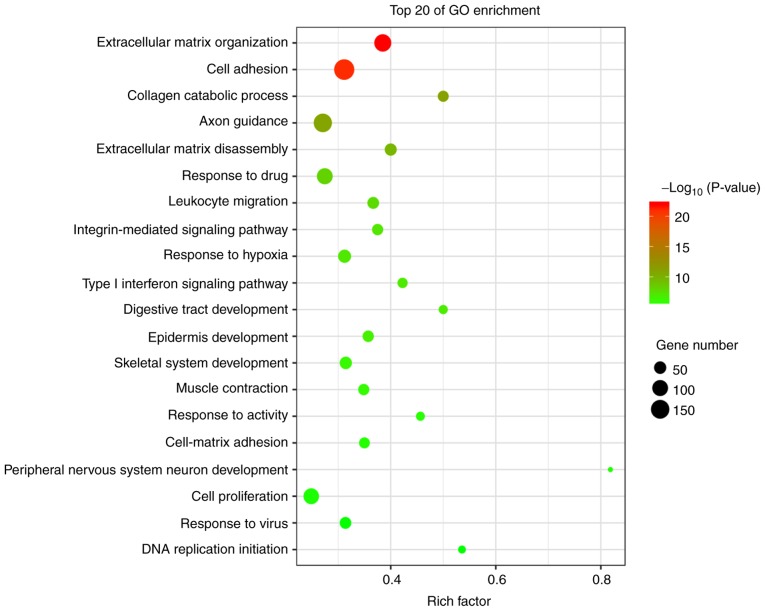
Top 20 GO enrichment terms of differential genes. The point size indicates the number of differentially expressed genes in that gene class, and the color indicates the enrichment effect. The abscissa is the enrichment factor, and the larger the number, the greater the enrichment degree. GO, Gene Ontology.

The genes that were differentially expressed in tongue cancer and paracancerous tissues were analyzed. GO analysis uncovered significantly enriched terms associated with three items: i) Biological processes: Extracellular matrix (ECM) organization, cell adhesion, collagen catabolic processes, ECM disassembly and the integrin-mediated signaling pathway; ii) molecular functions: Calcium ion binding, integrin binding, growth factor activity and ECM structural constituents; and iii) cellular components: Proteinaceous ECM, extracellular region and ECM. The most significantly enriched terms were analyzed as the ECM organization and cell adhesion (Figs. 4 and 5).

#### KEGG analysis

**Figure 6.**
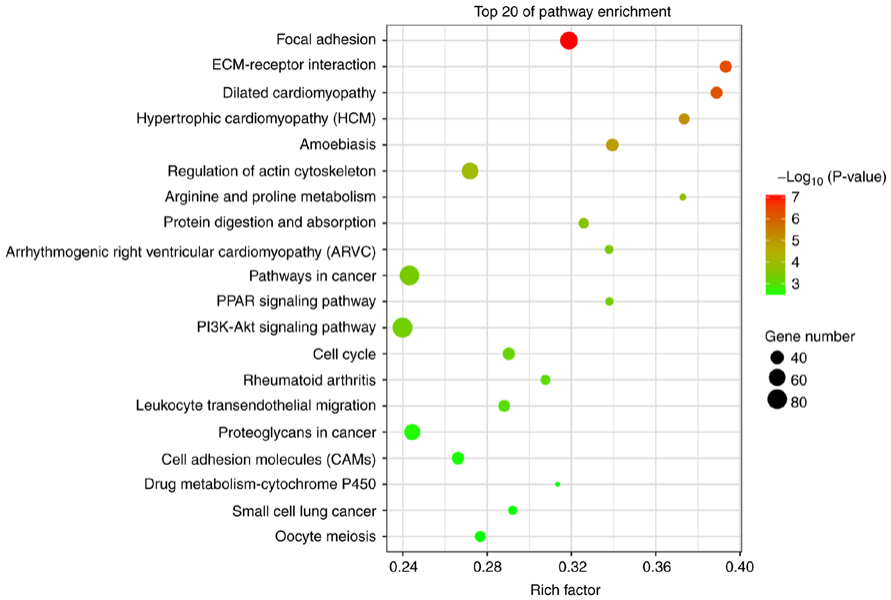
Kyoto Encyclopedia of Genes and Genomes enrichment for differentially expressed genes.

Of the pathways identified by KEGG pathway annotation and analysis, 61 were significantly downregulated and 43 were upregulated (Fig. 6). The most differentially activated pathways were focal adhesion, ECM-receptor interaction, pathways in cancer, small-cell lung cancer, phosphoinositide 3-kinase (PI3K)-Akt signaling and cell adhesion molecules (CAMs).

#### RT-qPCR

**Figure 7.**
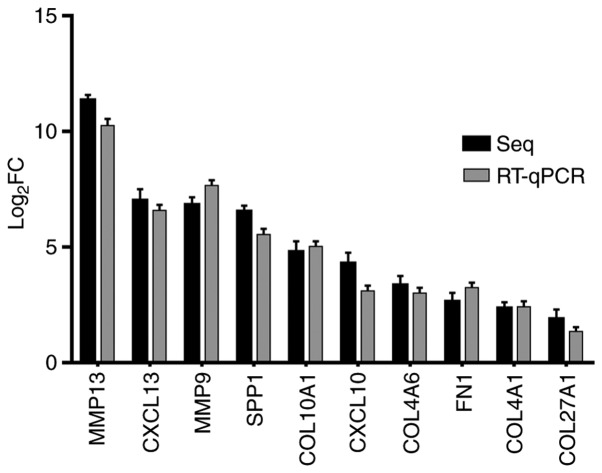
Gene expression changes by RT-qPCR. Changes of genes expression were shown in multiples. There were no significant differences in the trend of gene expression changes between RT-qPCR and RNA-seq. RT-qPCR, reverse transcription-quantitative PCR.

The differences of fold changes were compared between RNA-seq and RT-qPCR. The results are shown as Fig. 7. These 10 genes were all upregulated in cancer tissues, and there was no significant biological difference between RT-qPCR and RNA-seq, indicating that the RNA-seq results were consistent with those of RT-qPCR.

#### Signaling pathway network

**Figure 8.**
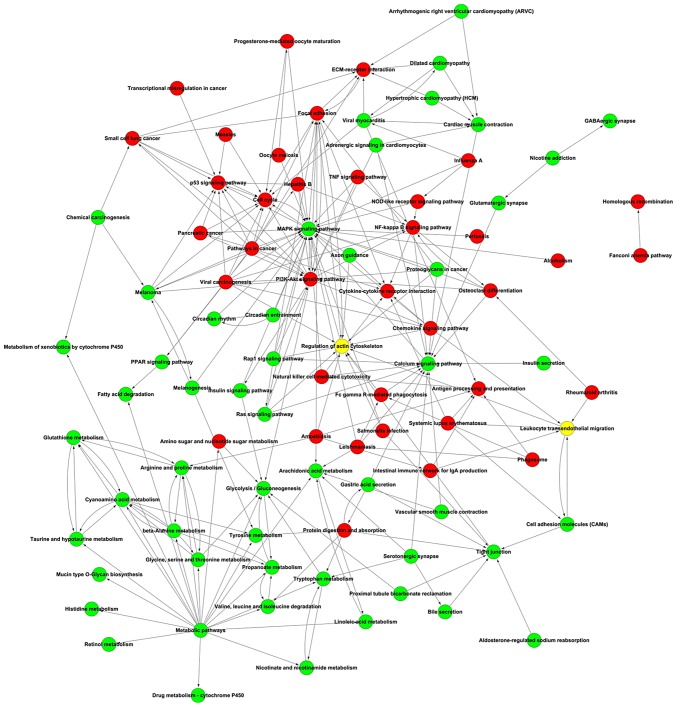
Kyoto Encyclopedia of Genes and Genomes network. Red circles, upregulation; green circles, downregulation; and yellow circles, both upregulation and downregulation in these signaling pathways.

Potential interactions among the differentially expressed pathways were depicted in the pathway network, including mitogen-activated protein kinase signaling, PI3K-Akt signaling, cancer, calcium signaling and focal adhesion pathways (Fig. 8).

## Discussion

Tongue cancer is a prevalent malignant disease that is difficult to diagnose in its early stages and is characterized by high rates of metastasis and postoperative recurrence (4). Hence, the molecular mechanisms implicated in tongue cancer must be elucidated to improve the clinical outcomes of affected patients. In the complicated pathological network of tumorigenesis, abnormal upregulation or downregulation of a single gene cannot adequately illustrate complex transcriptome changes during tumor development. Consequently, GO and KEGG pathway annotations were used in the present study to analyze the transcriptome data of tongue cancer.

GO analysis uncovered significantly enriched terms associated with ECM organization (upregulated), cell adhesion (downregulated) and collagen catabolic processes (upregulated). KEGG analysis demonstrated that these differentially expressed genes were mainly enriched in the focal adhesion pathway (upregulated), ECM-receptor interaction pathway (upregulated), PI3K-Akt pathway (upregulated) and CAMs (downregulated). RT-qPCR was used to verify the sequencing results.

The occurrence, progression and metastasis of tongue cancer are closely linked to the ECM (14). Proliferation and fibrosis of connective tissues have been reported to accompany tumorigenesis and progression of solid tumors (15,16). Upregulated type I collagen, fibronectin (FN1) and other ECM proteins in breast cancer (17,18) and upregulated collagens, non-collagen glycoproteins and proteoglycans in hepatocellular carcinoma (19) suggest that the expression and component changes in the ECM are crucial indicators of tumor progression. Our RNA-Seq results revealed decreased elastin assembly and increased collagen degradation in tongue cancer tissues. Current evidence has demonstrated that fibrous tissue hyperplasia is a protective response of the body, which may be used as a prognostic indicator (20). Abnormal deposition and increased ECM rigidity are apparent in fibrotic and malignant cancer tissues (21). Neovascularization is a hallmark of cancer. The collagen (COL) gene family encodes collagen in the ECM, which can participate in inducing angiogenesis in cancer (22–24). Sequencing and qPCR results demonstrated that COL4A1 and COL4A6 were significantly upregulated in tongue cancer, suggesting that these genes may play important roles in the occurrence and development of tongue cancer. Matrix metalloproteinases (MMPs) are important enzymes in the ECM, which can degrade the basement membrane and ECM and promote tumor cell invasion and metastasis (25–27). MMP-9 and MMP-13 were found to be significantly upregulated in tongue cancer, further confirmed this function.

The mammalian ECM consists of ~300 proteins (28). The ECM acts as a structural support and infiltration mediator for tissues and organs, and as a cellular signal mediator that regulates cell phenotypes by transmitting signals via membrane surface receptors. A relevant study on hepatocellular carcinoma demonstrated that prostaglandin E2 (PGE2) activates prostaglandin EP3 receptor (PTGER3) in the mesenchymal cells surrounding tumor cells, thereby promoting the activation and release of vascular endothelial growth factor, MMP-2 and MMP-9; in this manner, PGE2 ultimately promotes angiogenesis and tumor cell growth (29). In addition, PTGER3 was found to regulate prostate cancer cell growth by targeting androgen receptors (30). KEGG analysis revealed that several ECM-receptor interactions were significantly upregulated and enriched. As depicted in the KEGG network, the ECM-receptor interaction pathway was directly linked to pathways in cancer, namely the small-cell lung cancer pathway, the PI3K-Akt pathway and CAMs in tongue cancer tissues. The interaction between the ECM and cell membrane receptors is considered to play a key role in tongue cancer development, suggesting that blocking such an interaction may suppress tongue cancer development and metastasis. Chemokines are cytokines that mobilize cells via chemotaxis. It was previously demonstrated that CXCL10, CXCL13 and other chemokines are closely associated with the occurrence and development of cancer (31,32). The sequencing and PCR results in the present study confirmed that the expression levels of CXCL10, CXCL13 and other chemokines in tongue cancer tissues were significantly increased, suggesting their importance in tongue cancer.

The PI3K-Akt pathway is widely distributed in various cells and is known to regulate cellular behavior, protein synthesis and angiogenesis (33). Under pathological conditions, dysregulation of the PI3K-Akt pathway may trigger cancer occurrence and progression (34). Three mechanisms are considered to be responsible for the biological functions of the PI3K-Akt pathway in cancer. First, the PI3K-Akt pathway suppresses apoptosis and stimulates cell proliferation. p-Akt can suppress the mitochondrial apoptotic pathway by downregulating caspase-9 (35). p-Akt can also inhibit cell apoptosis and promote proliferation by activating glycogen synthase kinase 3β, FOX proteins, the MDM2 protooncogene and the transcription factor NF-κB (36). Second, the PI3K-Akt pathway regulates cell cycle progression. By transmitting the mitotic signal to p70s6k, the PI3K-Akt pathway upregulates the expression of cell cycle-related proteins and CDK4. Subsequently, the upregulated CDK4 inhibits downregulation of p21Cip1/WAF1 and p27Kip1, thus triggering the progression from the G_1_ to the S phase (37). Finally, the PI3K-Akt pathway stimulates tumor angiogenesis and tumor cell migration by upregulating the hypoxia-inducible factors, nitric oxide and cyclooxygenase 2. After activating the PI3K-Akt pathway, downregulated E-cadherin, which is regulated by phosphorylated glycogen synthase, inactivates intercellular adhesion molecules and enhances the metastatic ability of tumor cells (38).

CAMs are functional molecules that mediate the contact and binding between cells, or between cells and the ECM. CAMs exert their biological effects via receptor-ligand binding, and participate in physiological and pathological processes, including cell proliferation, differentiation, movement, immune and inflammatory responses, coagulation, and cancer cell metastasis. Generally, CAMs comprise the integrin family, the immunoglobulin superfamily, the selectin family, the cadherin family, and other adhesion molecules. Epithelial-to-mesenchymal transition (EMT) usually occurs during tumor progression and embryonic development, leading to transformation of cells from an epithelial phenotype with strong adhesions to a mesenchymal phenotype with invasive ability and increased motility (39). EMT causes weakening of intercellular connections in cadherin-based isotypes and attenuates the ability of cells to anchor to the cytoskeleton via cadherin-catenin. Subsequently, activated CAMs with weak adhesion or heterotypic CAMs induce detachment of *in situ* tumor cells and entry into the blood or lymphatic circulation. CAMs regulate cell-cell adhesion and interactions between cells and the surrounding microenvironment. Moreover, CAMs and their relevant pathways play important roles during different stages of tumor progression (40).

Focal adhesions (FAs) are the main mediators of the connection between cells and the ECM. FAs induce enhanced tumor cell motility and EMT through integrin-based signaling transition and mechanical structural support. Upregulation of genes in the FA pathway is closely associated with tongue cancer progression. During malignant progression from atypical dysplasia of the oral mucosal epithelium into oral squamous cell carcinoma, focal adhesion kinase (FAK) is gradually upregulated. Thus, FAK may be used as a diagnostic marker for precancerous oral epithelial lesions (41). In FAK^−/−^ mice, the rate of papilloma formation decreased by 50%; once benign tumors had formed, loss of FAK inhibited malignant progression (42). Blocking the FAK pathway was shown to markedly decrease cell adhesion ability and cell invasion and motility in head and neck squamous cell carcinoma (43). FAK is upregulated in most tumors, and its level is associated with the malignant behavior of the tumors. FAK levels are markedly higher in malignant metastatic tumor tissues compared with those in normal tissues or invasive tumor tissues (44). A relevant clinical trial reported that FAK expression levels are inversely correlated with the survival of patients with tumors (45). Therefore, FAK may be a potential diagnostic marker for early-stage tongue cancer, and FAK receptors may represent promising therapeutic targets. In the present study, FN1 was found to be highly expressed in tongue cancer tissues. In addition, secreted phosphoprotein 1 (SPP1; also referred to as osteopontin), is a secreted phosphorylated glycoprotein involved in cell adhesion, proliferation, migration, inflammation, immune response and signal transduction, and can induce new angiogenesis (46,47). The present study demonstrated that SPP1 was significantly upregulated in tongue cancer, suggesting that FN1 and SPP1 play important roles in the development of tongue cancer.

Collectively, the activity of the FAK, ECM-receptor, PI3K-Akt and CAM pathways varies greatly during tongue cancer occurrence and progression. The activated PI3K-Akt pathway stimulates cell proliferation, alters the cell cycle, inhibits apoptosis and induces angiogenesis. Phosphorylated glycogen synthases further downregulate E-cadherin and, thus, block the CAM pathway. Impaired CAMs result in compositional changes in the signaling molecules in the ECM, which, in turn, cause alterations in the relevant pathways. In addition, a unique microenvironment affected by component and rigidity alterations of the ECM further affect tumor cell metastasis. In summary, the progression, invasion and metastasis of malignant tumors is a dynamic process. The interaction between the PI3K-Akt pathway and CAMs was shown to trigger changes in the ECM, thus further aggravating an unfavorable microenvironment in tongue cancer.

In the present study, potential molecular mechanisms and tumor-related pathways during tongue cancer progression were analyzed through RNA-Seq. However, certain limitations should be addressed. First, the small sample size may have led to individual bias. Since transcriptome sequencing is a sensitive detection method, our sequencing data only provided referential information. Second, differentially expressed pathways were abundant, and some were significant during tongue cancer progression. The critical pathways and underlying mechanisms of tongue cancer require further investigation.

In conclusion, transcriptomes from four tongue cancer tissues and four paired paracancerous tissues were analyzed, and 1,700 upregulated and 2,249 downregulated genes were identified. These differentially expressed genes were mainly enriched in the FA, ECM-receptor interaction, PI3K-Akt and CAM pathways. These pathways synergistically promoted tongue cancer occurrence and progression, and may be potential biological markers and therapeutic targets for early-stage tongue cancer. However, due to the small sample size and sensitivity of RNA-Seq, further molecular biology research is required to elucidate the roles of differentially expressed pathways in tongue cancer progression, in order to provide a wider theoretical and experimental basis for the clinical diagnosis, treatment and prognosis of patients with tongue cancer.

## Supplementary Material

Supporting Data

## Figures and Tables

**Table I.** Basic patient information.

Case no.	Age, years	Sex	Stage	Differentiation	Type
1	66	Male	T3N2bM0, IVb	Well-differentiated	Ulcerative infiltrating
2	73	Male	T2N0M0, II	Well-differentiated	Exogenous
3	57	Male	T3N2bM0, IVb	Moderately differentiated	Ulcerative infiltrating
4	73	Female	T2N2cM0, IVb	Well-differentiated	Ulcerative infiltrating

**Table II.** Primers used in reverse transcription-quantitative PCR.

Gene	Primers (5′-3′)
COL4A6	F: CTAACTATCTAAGGGGCTGTGC
	R: ATTGGCTGATGGTGAGATTTGTATC
SPP1	F: AGAAGTTTCGCAGACCTGAC
	R: TTTCAGCACTCTGGTCATCC
FN1	F: TACCATCAGAGAACAAACACTAATG
	R: AAGAACTCTAAGCTGGGTCTGC
MMP13	F: TCTGGACAGACTGGCTGTTG
	R: TTGAAGGGATGTGATGGTCA
CXCL13	F: CTCTGCTTCTCATGCTGCTG
	R: CAGCTTGAGGGTCCACACAC
MMP9	F: TTCAGGAGACGCCCATTTC
	R: GTCGTCGGTGTCGTAGTTGG
COL10A1	F: CATGCCTGATGGCTTCATAAA
	R: AAGCAGACACGGGCATACCT
COL27a1	F: GGAACGGACAGGTCTTTGAA
	R: GGGTCCGGAAGGTGAATAGT
COL4A1	F: GAACGGGCCCATGGACAGGACTTG
	R: AGGTGGACGGCGTAGGCTTCTTG
CXCL10	F: GTACGCTGTACCTGCATCAGCATTAG
	R: CTGGATTCAGACATCTCTTCTCACCC

F, forward; R, reverse; COL, collagen; FN1, fibronectin; SPP1, secreted phosphoprotein 1; matrix metalloproteinase.

**Table III.** Top 10 differentially expressed genes among mRNAs and non-coding (nc)RNAs.

A, mRNAs		

Gene ID	log_2_FC	Up/downregulation
KRTAP13-2	−10.52	Down
KRT36	−10.07	Down
KRTAP13-1	−9.95	Down
MYOC	−9.26	Down
KRT84	−8.96	Down
CA9	9.07	Up
IL24	9.27	Up
MMP10	9.85	Up
S100A7A	10.00	Up
MMP13	11.35	Up

**B, ncRNAs**		

**Gene ID**	**log_2_FC**	**Up/downregulation**

RMST	−8.62	Down
LOC105375180	−7.20	Down
DIO2-AS1	−6.83	Down
LOC105372641	−6.82	Down
TATDN2P3	−6.78	Down
LINC00520	6.83	Up
LOC101928272	7.26	Up
AFAP1-AS1	7.33	Up
RFTN1P1	7.47	Up
LINC01322	8.86	Up

FC, fold-change.

## Availability of materials and data

All data generated or analyzed during the present study are included in this published article.
