# Supplementary material for: Transcriptome analysis of tongue cancer based on high-throughput sequencing
Source: Oncol Rep. 2020 Mar 23;43(6):2004–16. doi: 10.3892/or.2020.7560 (PMC7160550; doi:10.3892/or.2020.7560)
Supplement: Supporting Data [file Supplementary_Data.pdf]

Table SI. Basic information of the patients.

Case no.	Age, years	Sex	Stage	Differentiation
1	66	Male	T3N2bM0	Well-differentiated
2	73	Male	T2N0M0	Well-differentiated
3	57	Male	T3N2bM0	Moderately differentiated
4	73	Female	T2N2cM0	Well-differentiated
5	67	Female	TNM0	Poorly differentiated
6	53	Female	T4aN2cM0	Poorly differentiated
7	62	Female	TNM0	Well-differentiated
8	59	Male	T3N0M0	Well-differentiated
9	57	Male	T2NM0	Moderately differentiated
10	65	Female	T1N2M0	Well-differentiated
11	51	Male	T2N2bM0	Well-differentiated
12	68	Male	T1N0M0	Well-differentiated
13	53	Female	T2N2bM0	Well-differentiated
14	57	Male	T3N2bM0	Well-differentiated
15	64	Male	T2N0M0	Well-differentiated
16	70	Male	T3N2bM0	Well-differentiated
17	52	Male	T2N0M0	Well-differentiated
18	64	Male	T2N0M0	Poorly differentiated
19	62	Male	T2N2cM0	Well-differentiated
20	59	Female	T3N2bM0	Well-differentiated
